# Ameloblastic fibro‑odontoma in the posterior mandible: A case report

**DOI:** 10.3892/mi.2023.123

**Published:** 2023-11-20

**Authors:** Mahima Goel, Ali Qamar, Mimansa Daftary, Sujata Rajesh Chhabile, Shruti Pundkar, Manish Sharma

**Affiliations:** 1 Department of Oral and Maxillofacial Surgery, Pacific Dental College and Hospital, Udaipur, Rajasthan 313001, India; 2 Department of Oral and Maxillofacial Surgery, Teerthankar Mahaveer Dental College and Research Institute, Moradabad, Uttar Pradesh 244001, India; 3 Department of Public Health Dentistry, JMF's ACPM Dental College, Dhule, Maharashtra 424001, India; 4 Department of Public Health Dentistry, Vidarbha Youth Welfare Society Dental College, Amravati, Maharashtra 444601, India; 5 Department of Oral Pathology, JMF's ACPM Dental College, Dhule, Maharashtra 424001, India

**Keywords:** ameloblastic fibro-odontoma, mixed odontogenic tumor, posterior mandible, enucleation, benign tumor

## Abstract

Ameloblastic fibro-odontoma (AFO) is a rare, slow-growing neoplastic lesion classified as a benign, epithelial odontogenic mesenchymal tumor. This tumor exhibits histological features characteristic of both ameloblastic fibromas and complex odontomas. The clinical manifestation of AFO is typically characterized by the asymptomatic enlargement of the jawbones. Radiographically, it presents as a distinct radiolucent region, indicating the presence of radiopaque substances with varying degrees of irregularities in size and morphology. Standard therapeutic intervention involves enucleation. Despite its benign nature, AFO can cause significant morbidity if left untreated. Therefore, prompt diagnosis and appropriate management are essential to ensure optimal patient outcomes. The present study describes the case (clinical presentation and management) of an 18-year-old male patient with an AFO lesion located in the posterior mandible. This particular case was treated with conservative measures involving surgical enucleation along with the extraction of the impacted tooth and the curettage of residual bone.

## Introduction

Ameloblastic fibro-odontoma (AFO) is an infrequent, gradually progressing, expansile epithelial odontogenic neoplasm (1). The aforementioned lesion exhibits histological characteristics and biological features similar to those of an ameloblastic fibroma (AF). However, in AFO, one or more cellular foci of inductive epithelium persist during the differentiation process, leading to the production of both enamel and dentin (2). According to the 2017 publication of the World Health Organization (WHO) classification, AFO should now be classified as an odontoma rather than an odontogenic tumor (3). It typically manifests in younger individuals and displays no significant tendency toward either sex. It has been observed to occur with an equal frequency in both the maxilla and mandible, and its commonly observed site of occurrence is in the region posterior of the premolars and molars (4). AFO, which frequently affects young children, is often challenging to diagnose prior to surgical intervention due to the lack of pre-surgical pathological information (1). This is typically resolved by relying on observations obtained from a presurgical biopsy performed under local anesthesia. Consequently, clinical diagnosis assumes an exceedingly prominent role in the preemptive delineation of surgical procedures, particularly as regards the determination of the feasibility of conserving teeth situated in close proximity to the lesion (5).

The present study describes the clinical case of an 18-year-old individual with AFO, with a lesion involving the posterior mandible that was managed through enucleation.

## Case report

An 18-year-old adolescent male was referred to the Department of Oral and Maxillofacial Surgery, Teerthankar Mahaveer Dental College and Research Institute, Moradabad in March, 2020 with a primary concern of swelling on the right side of his face. The patient's medical history indicated the existence of an indistinct, non-painful and slowly developing bulge over a span of 1 year. Moreover, a discernible slight asymmetry of the face was noted and linked to the engagement of the posterior area of the mandibular bone. The medical records of the patients did not reveal any significant findings, and the dental history did not indicate any evidence of local injury or infectious activity at the site of the lesion. Upon an extraoral examination, it was determined that the enlargement exhibited an osseous morphology, characterized by a rigid consistency, with the integumentary covering displaying typical characteristics. No paralysis was identified in the affected area, although the displacement of the inferior alveolar nerve was detected. During an intraoral examination, an asymptomatic protuberance was observed on the posterior aspect of the right mandible. The mucosal layer exhibited unremarkable pigmentation, which was consistent with the overlying skin. The swelling spanned from the second to third molar region and displayed buccal and lingual cortical expansion.

**Figure 1:**
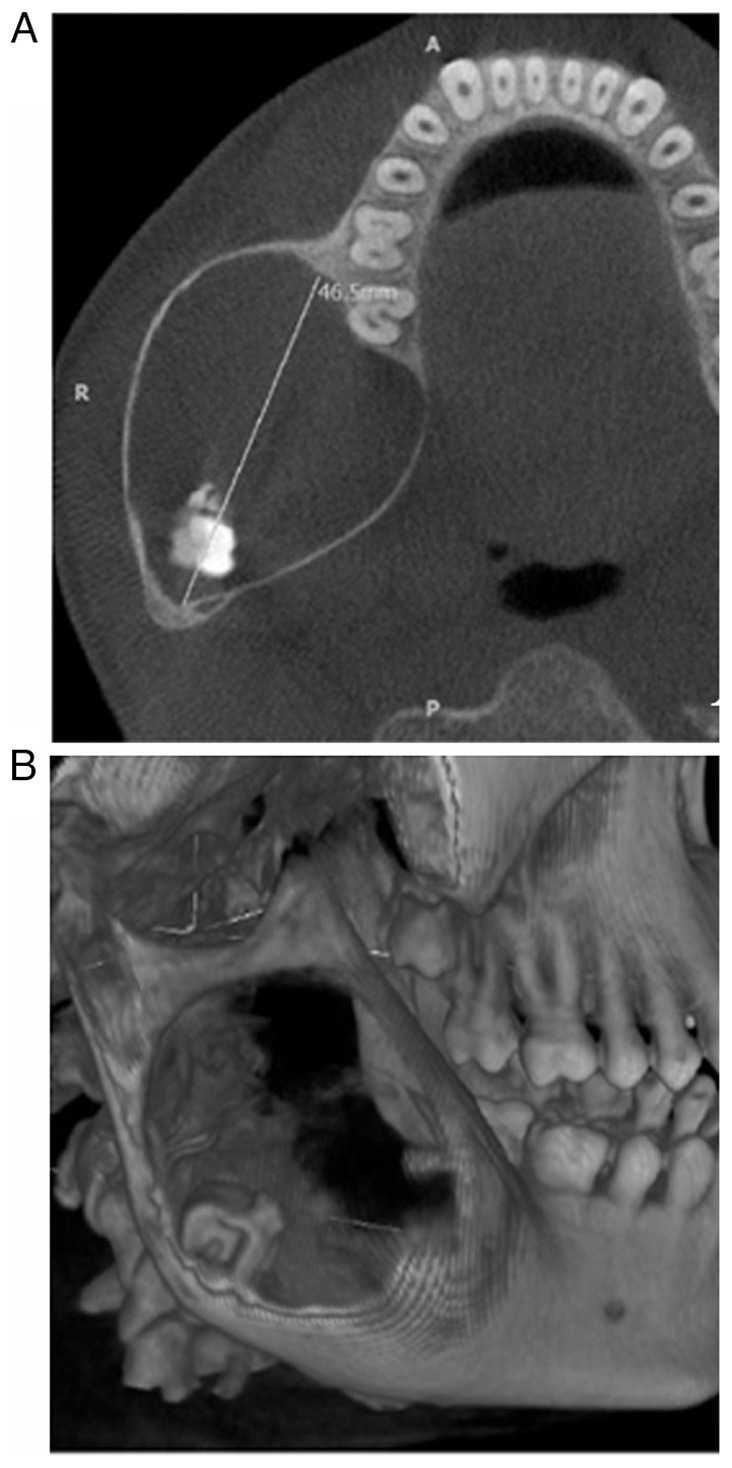
(A) Computed tomography image illustrating the anteroposterior expansion of the lesion, extending anteriorly from the lower right first molar region to the posterior border of the ramus. A, anterior; R, right; P, posterior. (B) An apically displaced third molar in the right mandibular region near the angle of the mandible.

Cone-beam computed tomography was used to identify a predominantly hypodense area of unilocular nature. This area involved the right body, angle and ramus of the mandible, with a thin corticated margin. The area extended anteriorly from the lower right first molar region to the posterior border of the ramus. The dimensions of this area were 46.5 mm anteroposteriorly, 38.3 mm mediolaterally, and 53.8 mm superioinferiorly, as illustrated in Fig. 1A. An apically displaced third molar in the right mandibular region near the angle of the mandible was observed, in association with calcification flecks (Fig. 1B). The presence of a bony crypt related to the third molar was not observed. However, both the lingual and buccal cortical plates appeared to be attenuated and displayed an obvious expansion without any discernible disruption of continuity.

**Figure 2:**
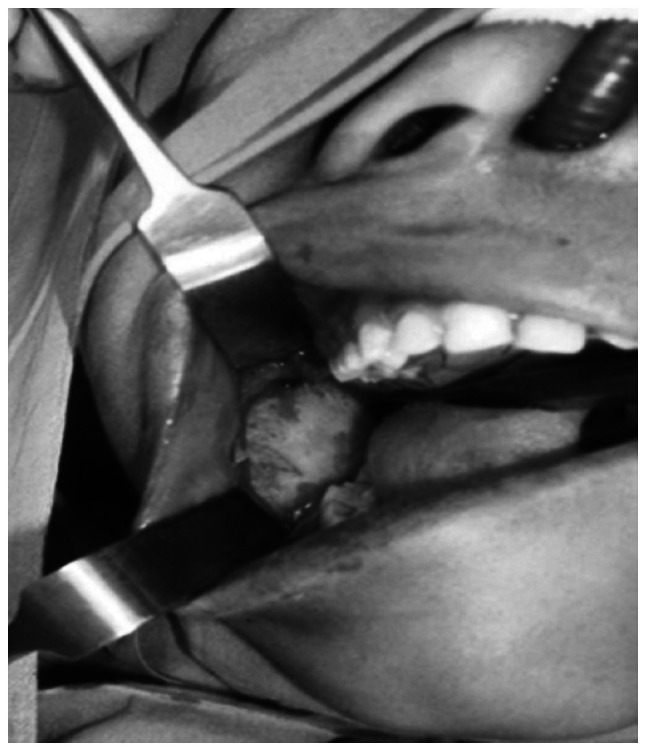
Surgical enucleation of the lesion with an impacted third molar.

Based on a comprehensive clinical and radiographic assessment, a tentative diagnosis of odontoameloblastoma, calcifying cystic odontogenic tumor, complex or compound odontoma, ameloblastic fibroma (AF), or ameloblastic fibro-dentinoma (AFD) was made. Following a scrupulous pre-surgical evaluation, the procedure was performed under general anesthesia, with an intraoral approach selected for the enucleation of the lesion along with the third molar. The extraction of the right second mandibular molar was performed due to root resorption. The curettage of the bony walls was subsequently performed, as illustrated in Fig. 2. Bone smoothing was accomplished, and the excision site was securely sutured with 3-0 vicryl sutures. Intermaxillary fixation was performed to immobilize the mandible and prevent any pathological fracture of the mandibular body.

**Figure 3:**
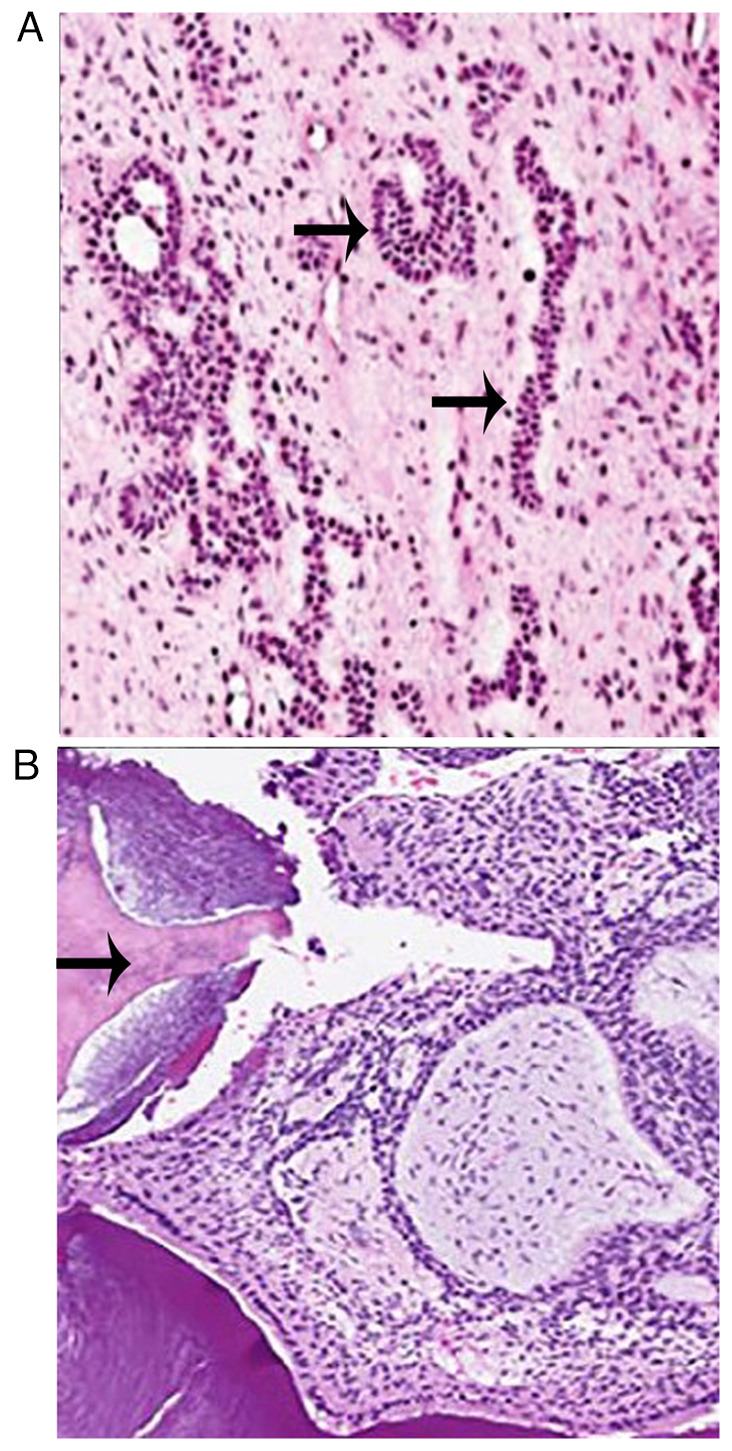
Histopathological image illustrating (A) an island of ameloblast-like cells (magnification, x40, and (B) a calcified mass in the hypocellular stroma (magnification, x40).

The tissue obtained from the lesion underwent a histopathological examination. Tissue was sent to pathology laboratory JMF's ACPM Dental College Dhule in 10% formaldehyde fixative solution and stored at 4˚C in the laboratory prior to the processing of tissue. Paraffin-embedded 5-µm-thick sections were stained with hematoxylin and eosin stain (Merck KGaA) at room temperature following Bancroft's protocol (6). The microscopic examination of the stained section under a binocular research microscope (Labomed LX500) revealed islands, cords and strands of ameloblast-like cells in a loose, immature connective tissue similar to dental papillae. Calcified flecks were also observed in the stroma near the aforementioned islands, with stromal calcifications indicating the possibility of dentinoid or enameloid material (Fig. 3).

**Figure 4:**
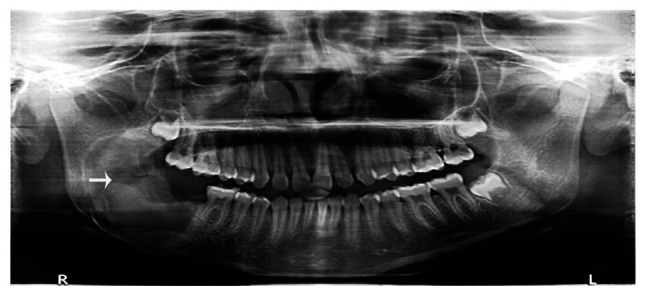
Post-operative evaluation of the site of the lesion (arrowhead), illustrating uneventful healing after 1 year.

The final diagnosis of the tumor was AFO. The soft tissue healing process was uneventful, with no evidence of recurrence during the 2-year follow-up period (Fig. 4).

## Discussion

AFO is a pathological condition that can be categorized as a slowly developing benign, mixed odontogenic neoplasm (1). This type of tumor is predominantly observed in the jaw bones, with a low incidence rate of 1-3% (4). The available literature substantiates that AFO predominantly targets juveniles, who have a mean age of 9.4±3.5 years, and evinces a predilection towards males, indicating a male-to-female ratio of 2.3:1(7). Furthermore, it is notable that the condition primarily affects the mandible, which differs from the conclusions presented in the 2005 WHO classification (8). The assertion made by the latter posited the absence of sex predilection in the distribution of the lesion, which was equally distributed across the mandible and maxilla, particularly in the molar region (7). Notably, several instances of the lesion were associated with an unerupted tooth or with the corresponding tooth displaced in the apical direction. This observation suggests that the origin of the lesion is tissues of dental follicle, primarily dental lamina (9).

According to the 2022 WHO classification of odontogenic tumors, a revision from the 2017 WHO classification, AFO has been designated as a precursor of complex odontoma (7). However, Soluk-Tekkesinand Vered (10) analyzed a series of cases of AFO and odontomas, and concluded that AFOs affect individuals between the ages of 3 and 16 years, and possess a size >2 cm. Bycontrast, odontomas tend to affect older individuals, whose age ranges ~27 years, and whose size is <2 cm. Hence, it is imperative to consider AFO with measurements >2 cm as a true neoplasm rather than a hamartomatous odontoma (10). The radiographic features of the AFO describe a distinct and identifiable radiolucency that was well-circumscribed and round-to-ovoid in shape. This radiolucency is surrounded by a thin sclerotic margin that contains various amounts of radiopaque material with an irregular size and form. Notably, the ratio of radiopaque to radiolucent areas may differ from one lesion to another, indicating the uniqueness of each case (2). The present case was documented in an 18-year-old male who presented with an enlargement >5 cm in the posterior section of the mandible with flecks of calcification, indicative of AFO.

A histopathological examination can provide an accurate diagnosis for cases involving odontoameloblastoma, complex odontomas, calcifying epithelial odontogenic tumor (CEOT), AF and AFD due to their similar clinical and radiological features (1).

Odontoameloblastoma is a combination of complex odontoma and ameloblastoma with an invasive nature, as observed in classical ameloblastoma. Radiographically, odontoameloblastomasrepresent a unilocular radiolucency associated with impacted molars and radiopaque substances. They may exhibit theperforation of cortical plates, which is not a feature of AFO. Histologically, odontoameloblastomas exhibita mature stroma with epithelial islands of ameloblastic cells and calcification (11).

There is a current and ongoing debate regarding the consideration of AF, AFO, AFD and odontomas as distinct entities or varying stages in the developmental process of odontomas (10). According to the 2017 WHO classification, certain lesions that exhibit a histopathological similarity to an AFO are likely indicative of the development of odontomas (3). Furthermore, it is plausible that the histopathological appearance of an AFO may be indistinguishable from that of developing odontomas. In such instances, the evaluation of concurrent clinical and radiological features may be a valuable tool for establishing diagnostic differentiation.

Histopathologically, it is imperative to note that a CEOT is typified by the noticeable presence of epithelial cells, homogenous eosinophilic amyloid-like material and calcification. Epithelial cells are organized, forming nests and sheets that are polygonal in shape and structure. In addition, the cells have a clear to eosinophilic cytoplasm and vesicular nuclei with prominent nucleoli (12). However, it is noteworthy that the aforementioned features were absent in the case described herein.

Microscopically, AF comprises various strands and small islands of the odontogenic epithelium. However, unlike the enamel organ-like structures that are commonly observedin AFO, AF does not possess such structures. Moreover, the fibrous elements in AF may range from cellular to mature collagenous tissue. Despite this fact, it is noteworthy that the primitive dental papilla-like appearance isnot obvious in AF (13). Furthermore, the ectomesenchymal component of AFO bears a striking resemblance to the dental papilla.

AFO shares some similarities with AFD; however, AFO contains both enamel and dentinal materials, whereas AFD solely comprises dentinal materials. Consequently, it can be noted that upon radiological observation, the characteristics of AFO entail a greater presence of opaque and denser elements within the lesion as opposed to AFD. The observable difference in radiology between AFD and AFO is distinctly evident by the multilocular nature of AFD lesions, which sets them apart from the unilocular lesions of AFO (14).

In terms of radiology and histology, unlike AFO, odontomas encompass a profound calcified component comprised of enamel, dentin, cementum and connective tissue pulp. The aforementioned components are observed in the advanced stages of compound odontoma, where oral maturation attains a state of completeness (10).

In the case described in the present study, the AFO exhibited a considerable size exceeding 5 cm and was well encapsulated with minimal inclination without localized invasion. Consequently, the recommended course of treatment involved conservative surgical intervention, entailing enucleation, in conjunction with the extraction of a non-erupted tooth. However, it is worth noting that documented cases exist in which the involved teeth have been preserved (15,16). Despite the limited propensity of the lesion for recurrence, there have been instances where conservative treatment aimed at preserving the associated teeth has yielded reports of recurrence (15). Consequently, to eliminate the possibility of recurrence, the unerupted third molar was extracted in conjunction with AFO enucleation.

In conclusion, the clinical and radiographic features of AFO are heterogeneous, and its diagnosis can be determined solely by a histological examination. Although infrequent, it is important to consider the possibility of its presence in the differential diagnosis of intraoral radiolucencies that comprise radiopaque material, particularly when managing pediatric patients. Irrespective of its classification, considering its benign behavior, minimal invasiveness and a low recurrence rate, the preferred method of treatment is a conservative approach, which typically involves enucleating the lesion and extracting the associated tooth to prevent future recurrences. The case described in the study had a positive post-operative course with no signs of recurrence. Owing to the potential for malignant transformation, the implementation of long-term monitoring and observation are it is strongly advised.

## Data Availability

The datasets used and/or analyzed during the current study are available from the corresponding author on reasonable request.
